# A case report of a lichenoid eruption in a young lady with Crohn’s disease

**DOI:** 10.1186/1479-5876-10-S3-P11

**Published:** 2012-11-28

**Authors:** Farida Benhadou, Véronique Del Marmol

**Affiliations:** 1 Dermatology, Erasme Hospital, Free University of Brussels, Belgium; 2 Erasme Hospital, Free University of Brussels, Belgium

## Background

Crohn’s disease is the most frequent inflammatory bowel disease. The use of the TNFα inhibitors has completely revolutionized the therapeutical management of recalcitrant cases of Crohn’s disease [1]. Unfortunately many side effects have been described in association with the use of the TNFα inhibitors[2]. The development of antinuclear antibodies is frequently described but is not always associated with a pathological condition [3]. The occurrence of lupus like syndrome after initiating the TNFα inhibitors therapy is rare and estimated with a prevalence of 0,5% to 1% [4]. We described a case of a lupus-like syndrome induced by the infliximab in a patient treated for Crohn’s disease.

## Case report

We report a case of a young woman suffering from Crohn’s disease since 2001. She was treated by infliximab at the dose of 5 mg/kg every 8 weeks since 2009.

**Figure 1:**
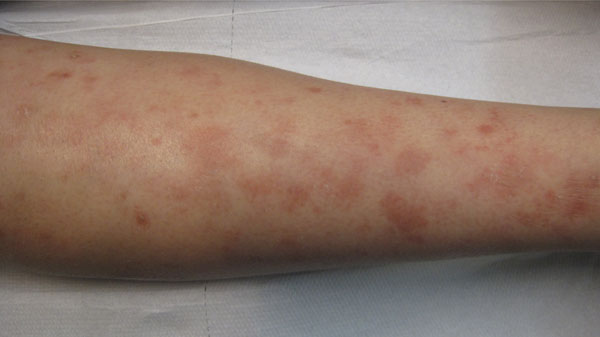


She developed in 2011 an extensive lichenoid eruption in her arms and legs [Figure 1]. She developed at the same time an important anemia. Her blood analyses showed the presence of antinuclear antibodies at a title of 1/640 and anti double strand DNA at a title of 25 UI that were negative before the initiation of the infliximab.

Skin biopsies were performed and showed an histological picture of lupus. Six weeks after the withdrawal of the infliximab therapy the skin lesions were completely healed and the anemia was resolved. A diagnosis of a lupus like syndrome induced by the infliximab was retained.

## Conclusions

Despite the frequent induction of autoantibody production in patients treated with TNFα inhibitors, the development of lupus like syndrome is uncommon, with a prevalence of 0.5% to 1%. The most cases have been described after the use of infliximab. The skin involvement is rare and the arthralgia are most frequent [5]. A resolution of the symptoms is observed after the discontinuation of the TNFα therapy.

Through the presentation of this case report, we wanted to discuss the role of this autoantibodies and their association with pathological conditions. We wanted also to focuse about the management of the lupus like syndrome induced by the TNFα inhibitors.
